# Egocentric and allocentric spatial memory in typically developed children: Is spatial memory associated with visuospatial skills, behavior, and cortisol?

**DOI:** 10.1002/brb3.1532

**Published:** 2020-03-14

**Authors:** Cristina Fernandez‐Baizan, Paula Nuñez, Jorge L. Arias, Marta Mendez

**Affiliations:** ^1^ Faculty of Psychology Instituto de Neurociencias del Principado de Asturias (INEUROPA) Oviedo Spain; ^2^ Faculty of Psychology Department of Psychology University of Oviedo Oviedo Spain; ^3^ Faculty of Medicine Department of Functional Biology University of Oviedo Oviedo Spain

**Keywords:** allocentric, children, cortisol, egocentric, spatial memory

## Abstract

**Introduction:**

Spatial orientation relies mainly on two frameworks. The egocentric depends on our own position and point of view. The allocentric relies on remembering, recalling, and recognizing environmental stimuli called landmarks. The aim of this study was to analyze the egocentric and allocentric spatial memory performance in children of different ages using two experimental memory card‐placing tasks. We also aimed to examine relationships between spatial memory and other cognitive, physiological, and behavioral factors that, potentially, could be associated with spatial memory performance. Those were other visuospatial functions, the regular behavior of the child, cortisol levels, and daily life spatial memory.

**Methods:**

We assessed 62 children (5, 6, and 7 years) using card‐placing tasks. We used RIST for IQ evaluation and subtest from NEPSY‐II for visuospatial ability assessment. Collection of saliva sample was carried out for cortisol analysis. Parents completed BASC questionnaire for behavioral evaluation and ECM‐Q questionnaire for daily life spatial memory evaluation.

**Results:**

Our results showed that older children performed better on mental rotation. Directionality, map interpretation, and daily memory were directly associated with both egocentric and allocentric orientation. Egocentric performance was positively related to leadership abilities but negatively to depression and atypicality, while allocentric performance was directly associated with adaptive behavior but inversely with hyperactivity. Finally, cortisol values were positively associated with allocentric performance.

**Conclusions:**

Our study shows the development of different spatial abilities between 5 and 7 years, as well as the relationship between orientation performance, visuospatial skills, behavior, and cortisol.

## Graphical abstract

The aim of this study was to analyze the egocentric and allocentric spatial memory performance in children of different ages using two experimental memory card‐placing tasks and to examine relationships between spatial memory and other cognitive, physiological, and behavioral factors that could potentially be associated with spatial memory performance, such as other visuospatial functions, the regular behavior of the child, cortisol levels, and daily life spatial memory. Our study shows the development of different spatial abilities between 5 and 7 years of age, as well as the relationship between orientation performance, visuospatial skills, behavior, and cortisol.

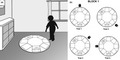

## 1 INTRODUCTION

To be able to orientate ourselves in our surrounding environment, we employ mainly two frameworks. The egocentric framework depends on our own organism's cues, in other words, our own position and point of view (Ruggiero, Iachini, Ruotolo, & Senese, 2009), considering information like turns, directions, distances, speed, etc. This egocentric framework also involves some other systems, like the kinesthetic, optic, and vestibular (van den Brink & Janzen, 2013). The allocentric framework relies on remembering, recalling, and recognizing environmental stimuli called landmarks (Ruggiero et al., 2009) that progressively compose a mental image about a place or location (Burgess, 2008).

Spatial orientation development starts early in childhood (see Fernandez‐Baizan, Arias, & Mendez, 2019a; Fernandez‐Baizan, Arias, & Mendez, 2019b for a complete review). While the allocentric framework has been widely studied in normalized developed children in recent years, the egocentric orientation has received less attention (Fernandez‐Baizan, Diaz‐Caceres, Arias, & Mendez, 2019). It is difficult, based on the current spatial orientation assessment tests available, to compare between the two frameworks and to establish a course of improvement for these abilities. There is consensus on that the egocentric information is the very first to be employed by infants (Acredolo, 1978; Acredolo & Evans, 1980), but there is no agreement about when allocentric framework is completely developed, finding some studies that at least half of 5‐year‐old children reach an adult‐level performance (Ribordy Lambert, Lavenex, & Banta Lavenex, 2017), while others consider that this happens between the ages of 7 and 10 (Leplow et al., 2003; Overman, Pate, Moore, & Peleuster, 1996; Ruggiero, D'Errico, & Iachini, 2016). For this reason, it is necessary to know how is the course of normalized development of spatial orientation in typical developed children. This will help to achieve an earlier diagnostic in risk populations, such as those affected by neurological conditions that have been found to suffer spatial orientation problems, such as the Williams syndrome (Nunes et al., 2013), cerebral palsy (Belmonti, Fiori, Guzzetta, Cioni, & Berthoz, 2015), fetal alcohol syndrome (Hamilton, Kodituwakku, Sutherland, & Savage, 2003), prematurity (Cimadevilla, Roldán, París, Arnedo, & Roldán, 2014), and developmental topographical disorientation (Palermo, Foti, Ferlazzo, Guariglia, & Petrosini, 2014).

However, spatial orientation performance may be influenced by numerous factors, such as gender, visuospatial abilities, hormonal regulation, and behavior pattern. Therefore, it seems important to characterize the execution on egocentric and allocentric spatial orientation taking into account its relationship with these factors. Adult men usually outperform adult women (Fernandez‐Baizan et al., 2019a, 2019b), although results in children are contradictory (Juan, Mendez‐Lopez, & Perez‐Hernandez, 2014; León, Cimadevilla, & Tascón, 2014; Rodriguez‐Andres, Juan, Mendez‐Lopez, Perez‐Hernandez, & Lluch, 2016; Sorrentino et al., 2019), and there is still no agreement on the influence of gender at earlier ages. It is also important to consider that in childhood the ability to orient ourselves in space is related to improvements in other cognitive functions. Previous studies have pointed out that visuospatial abilities such us mental rotation and the ability to transfer maps from 2D to 3D environments have been related to spatial memory abilities in both adults (Astur, Tropp, Sava, Constable, & Markus, 2004) and children (Vasilyeva & Bowers, 2006). Hormonal regulation has been proposed as an important factor on cognitive performance. Higher cortisol values seem to be related to main brain areas involved in this function, such as hippocampal volume in adults (Pruessner, Pruessner, Hellhammer, Bruce Pike, & Lupien, 2007), children, and adolescents (Wiedenmayer et al., 2006). However, there are still contradictory behavioral results in adults, with some studies pointing out that higher cortisol levels could improve spatial orientation performance (Kukolja, Thiel, Wolf, & Fink, 2008; Meyer et al., 2013), while others do not (Schwabe et al., 2007; Schwabe, Oitzl, Richter, & Schächinger, 2009). In childhood, moderate to high cortisol values have been related to improvement in some cognitive functions (Bäumler et al., 2014; Blair, Granger, & Razza, 2005; Davis, Bruce, & Gunnar, 2002; Forns et al., 2014; Saridjan et al., 2014), but to our knowledge, the association between this hormone, spatial orientation, and visuospatial abilities have not yet been studied in children. Finally, the way we behave on a day‐to‐day basis can also affect neuropsychological performance. In this sense, it has been found that children who present an externalizing behavioral pattern tend to present greater difficulties in attentional tasks and executive functions, while children who show a more internalizing pattern present greater problems in verbal abilities and in memory, while both behavioral profiles showed difficulties in visuospatial abilities (Blanken et al., 2017). Thus, spatial orientation performance may also be associated with child's behavior.

The main aim of this study was to analyze the egocentric and allocentric spatial orientation performance in typically developed children aged between 5 and 7 using functional and ecological tasks, which allow us to reproduce similar conditions that occur in daily orientation and to compare between frameworks, and to know how these abilities are related to gender, spatial cognition, behavior, and cortisol levels. First, our purpose was to examine whether or not there is an improvement of these frameworks at these ages. We hypothesized that the allocentric framework, but not the egocentric framework, would progress from the ages of 5–7. Second, we aimed to compare egocentric and allocentric orientation performance between genders. We hypothesized that boys would outperform girls in the allocentric test but would obtain similar results in the egocentric one. Third, we aimed to compare both types of orientation in order to know which is better performed. We hypothesized that egocentric achievement would be better than allocentric, as the first one develops earlier in childhood. Fourth, we aimed to relate our spatial orientation tasks with the performance of the child on daily life spatial memory, in order to verify whether our tasks are effectively functional and ecological. We hypothesize that day‐to‐day memory measurements and spatial orientation test results would be moderately associated. Fifth, our purpose was to examine the existence of any relationship between spatial orientation tasks and other visuospatial tests, with the aim to provide a more complete profile of space ability development. We expected that spatial orientation and visuospatial skills would be related, but with low to moderate magnitude, as they measure different functions. Sixth, we aimed to verify whether the behavior pattern is related to spatial orientation performance. We hypothesized that maladaptive behaviors will be related to worse execution in orientation, while adaptive behaviors, with better results. Seventh and last, we aimed to analyze the existence of any association between cortisol salivary levels and visuospatial and spatial orientation performance. We hypothesized that occasional higher levels of cortisol would be associated with visual and spatial function achievements.

## 2 MATERIALS AND METHODS

### 2.1 Participants

The sample was composed of 62 children aged 5 (*N* = 21), 6 (*N* = 21) and 7 (*N* = 20). Thirty were females. Children and parents, which were recruited from schools, primary care centers, and hospitals of Oviedo (Spain), were informed about the purpose of the study and provided written consent. Exclusion criteria included neurological, psychological, or physical conditions and disorders that could potentially interfere with the results. In addition to these criteria, children who obtain an IQ result lower than 85 (assessed with Reynolds Intellectual Screening Test (Reynolds & Kamphaus, 2003)) were not included. Thus, after eliminating four of 66 children that did not meet these criteria, the final sample consisted of 62 participants. This study was conducted in compliance with the European Community Council Directive 2001/20/EC and the Helsinki Declaration for biomedical research involving humans.

### 2.2 Instruments

#### 2.2.1 Egocentric Spatial Memory Task—Child version

**Figure 1:**
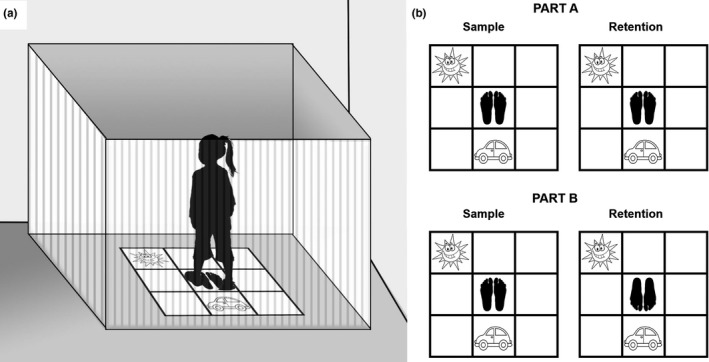
Egocentric Spatial Memory Test. (a) Representation of Egocentric Spatial Memory Test—Children version. (b) Example items of Egocentric Part A and Part B from Egocentric Spatial Memory Test. In Part A, child remains in same position in both sample and retention, but in Part B, child memorizes cards in one position in sample, but then he/she is rotated for retention trial

This is an adaptation of the Egocentric Spatial Memory Tasks for adults (Fernandez‐Baizan et al., 2019a, 2019b; Fernandez‐Baizan et al., 2019), based on Hashimoto's test for head disorientation assessment (Hashimoto, Tanaka, & Nakano, 2010). It consists of a square template (90 × 90 cm) placed on the floor and divided into a matrix of nine small squares (3 × 3, 30 × 30 cm each). Four opaque panels (180 × 180 cm) are surrounding the floor template and placed in the shape of a square, with the aim of avoiding any visual information that might interfere with the purely egocentric response. The child must stand in the central square of the matrix, and two picture cards (15 × 15 cm) are used as stimuli (a sun and a car) (See Figure 1a). This test examines the ability to represent spatial locations surrounding the child. It consists of two different parts: A and B. In Part A, while the child is standing in the central square of the template, he/she is asked to remember the location of two cards (car and sun), each placed randomly on one of the eight squares surrounding him/her. After ten seconds, the examiner removes the cards, hands them to the child, and orders him/her to put them back in their original position. In Part B, the child has to remember the same two cards' locations used in Part A. However, immediately after the two cards have been removed, the child and the examiner rotated to the right or left, 90 or 180° as determined in the test, and then, he/she is asked to place the two cards in the same places as before. During the memorization or sample phases, the examiner stood behind the child, but rotated with him/her in the changes of position, with the aim to avoid that the examiner became a static point of reference. In each part, A and B, the child undergoes five consecutive trials, scoring one point each time he/she places a card correctly. Therefore, scores vary between zero and ten points in each part (See Figure 1b).

#### 2.2.2 Allocentric Spatial Memory Task—Child version

**Figure 2:**
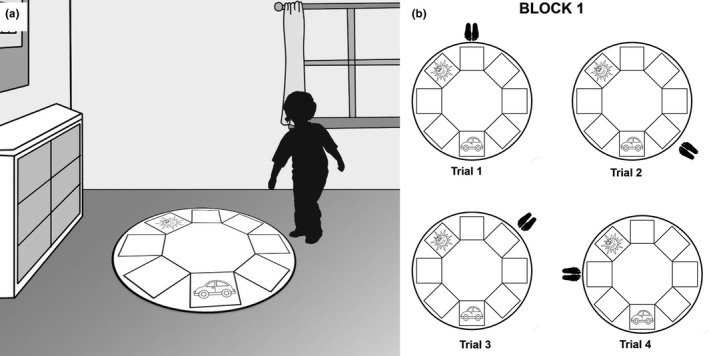
Allocentric Spatial Memory Test. (a) Representation of Allocentric Spatial Memory Test—Children Version. (b) Example of a Block from Allocentric Spatial Memory Test. In this Block 1, the location of the cards is always the same, but the position of the child varies in each trial (Trial 1, 2, 3, and 4)

An adaptation of the Allocentric Spatial Memory Task for adults was used. This test employs a circular template (65 cm diameter) placed on the floor. Along the perimeter are eight squares (18 × 18 cm) that can be used as possible locations. The same two picture cards (15 × 15 cm) are used as stimuli (a sun and a car). The use of the same stimuli in both orientation tasks has had the objective of avoiding that the results found in both tasks could be due to a possible preference of the child for other different stimuli, as well as to try to keep both tasks as methodologically similar as possible. The whole task is carried out in a rectangular room where objects and furniture remain visible for the child (See Figure 2a). This test examines the ability to represent spatial locations of objects using environmental information. The child stands in front of the circular template and he/she is asked to memorize the location of two cards (car and sun), each placed on one of the eight squares. After ten seconds, the examiner removes the cards and blindfolds the child, walking with him/her to a different point on the template. At this point, the mask is removed, and he/she is asked to put the cards back in their original position. Errors in the placement of the cards are corrected showing the right position, while correct answers are congratulated. The test consists of three blocks with four trials each, and the position of the cards is maintained for each block. The child obtains one point each time a card is placed correctly, and therefore, scores vary between zero and 24 points. The test ends either when child finishes the 12 trials (three blocks) or when he/she obtains zero points in two consecutive trials in the same block (stop criterion). (See Figure 2b).

#### 2.2.3 Reynolds Intellectual Screening Test (RIST)

This is a screening test (10–15 min) to estimate the intelligence quotient (IQ) for people between the ages of 3 and 94 (Reynolds & Kamphaus, 2003). It consists of two tasks: *Guess what* for verbal IQ assessment, where the examiner reads some riddles and definitions, and the child has to answer with the accurate word, and *Odd‐item* for nonverbal IQ assessment, where the child is shown several pictures and he/she has to point out the different or incongruent one.

#### 2.2.4 Geometric Puzzles (NEPSY‐II)

This subtest was employed to measure mental rotation abilities (Korkman, Kirk, & Kemp, 2007). The child is shown pages with a grid that contains several geometric figures inside and several geometric figures outside of the grid. He/she must pair two of the figures outside the grid with two of the figures inside. In this task, mental rotation skills are necessary when some of the figures outside and inside the square are equal, but they are not in the same position, that is, one of them has been rotated. This task includes 12 trials for 5‐ and 6‐year‐old children, and 20 for 7‐year‐old children.

#### 2.2.5 Route Finding (NEPSY‐II)

This subtest was employed for the directionality and spatial relation assessment, as well as to evaluate the ability to interpret a schematic map and be able to transfer this information to a more complex map (Korkman et al., 2007). The child is shown a schematic map with a target house and he/she is asked to find the house on a larger map, with more streets and more houses available. Children performed 10 trials in this task, unless they met the stop criterion (five consecutive erroneous trials).

### 2.3 Salivary sampling and cortisol assay protocols

Parents received and followed previous instructions required for the cortisol sample collection. The instructions were the following: no exercise in the previous two‐hour period; no eating, no gum or sweets, no drinking liquids except water, and no brushing teeth in the previous half‐hour period. Collection of the saliva sample took place at the end of the neuropsychological assessment and was collected with the Salivette device (Sarstedt, Germany). Both the child and examiner washed their hands, and he/she was asked to rinse his/her mouth with cold water. Then, the examiner introduced the cotton swab in child's mouth and asked him/her to chew the swab in order to stimulate salivation. After 60 s, the examiner returned the cotton to its tube. No saliva stimulants were used to encourage salivation. Samples were recorded between 16:45 and 18:20 p.m. and stored at −20°C until they were analyzed. The cortisol level was determined by an enzyme immunoassay method using the ELISA kit (Cortisol Competitive ELISA Kit; Thermo Fisher Scientific Inc.). The inter‐assay and intra‐assay variations were 8.1% and 8.8%, respectively. Assay sensitivity was 17.3 pg/ml.

### 2.4 Behavior Assessment System for Children—Parent version (BASC)

This is a questionnaire for parents that assess children's behavioral and emotional outcomes (Reynolds & Kamphaus, 1992). It is divided into different levels according to the child's age. In the present study, we used level one (3‐ to 6 year‐olds, preschool education) and two (6‐ to 12‐year‐olds, primary education) with 130 and 134 items, respectively. Each question describes a behavior that can be assessed in four levels according to its frequency of occurrence in the last 6 months (A: never; B: sometimes; C: frequently, and D: almost always). Two dimensions are considered in this questionnaire: adaptive and clinical. The adaptive and adjustment dimensions correspond to adaptability, social skills, and leadership behaviors, while the clinical or maladjustment dimensions include aggressiveness, hyperactivity, behavioral problems, attention problems, atypicality, depression, anxiety, shyness, and somatization behaviors.

### 2.5 Parent questionnaire of the Evaluación Clínica de la Memoria (ECM‐Q)

An adapted version of the *Evaluación Clinica de la Memoria (ECM‐Q)* was used by the selection of nine of its items (Juan et al., 2014). In this questionnaire, parents were asked about their child's spatial memory abilities in daily life. Each item is rated on a Likert scale from one to four (One—Never, Four—Always). Items included were as follows: (a) He/she remembers the path to go home, (b) he/she has good orientation, (c) he/she forgets how to go to one place, until he/she has been explained recently how to reach it, (d) he/she remembers where he/she has left their things, (e) he/she gets lost in known places, (f) he/she remembers were things are kept, (g) he/she recognizes places where he/she has been before, (h) he/she is used to getting lost in places where he/she has been before, and (i) he/she is good at learning the path to reach a new place.

### 2.6 General procedure

The experiment took place in the Faculty of Psychology and local primary schools (Oviedo, Spain). Children were tested individually by trained psychologists, starting with the Reynolds Intellectual Screening Test (RIST), Geometric Puzzles from NEPSY‐II, Route Finding from NEPSY‐II, Egocentric Spatial Memory Test—Child version, and Allocentric Spatial Memory Test—Child version. When the neuropsychological evaluation finished, cortisol salivary samples were collected. While children were performing the tests, parents completed sociodemographic, behavioral, and daily spatial memory questionnaires. The whole procedure lasted 60 min and was done in one unique session.

### 2.7 Statistical analysis

Analyses were performed with SPSS 19. Saphiro–Wilk was used to test normality and Levene tests were used to test normality and homogeneity. A two‐way ANOVA (Age × Gender) was applied. Repeated‐measures ANOVAs were used to compare Allocentric blocks and *t* tests were employed to compare egocentric parts A and B, and total Egocentric versus Allocentric performance. Multiple comparisons have been corrected by false discovery rate (FRD) (Q 5%) (Benjamini, Krieger, & Yekutieli, 2006). A bivariate Pearson correlation analysis was conducted to assess spatial orientation scores with other neuropsychological tasks and cortisol levels. The Cohen's *d* effect size was reported for significative comparisons (*d*). Differences were considered significant for *p* < .05.

## 3 RESULTS

### 3.1 Descriptive data

Mean characteristics of children and their families are presented in Table 1. Mother's age mean is 41.09 (±4.22) and father's age, 43.08 (±5.29). Mean and standard deviation of direct neuropsychological scores and cortisol values in terms of age are displayed in Table 2, as well as percentage of correct answers in Egocentric and Allocentric tasks. In Table 3, the same variables are grouped according to gender.

**Table 1:** Frequencies (%) of sociodemographic and descriptive characteristics of the sample and their parents

	Frequencies (%)	
Laterality	Right‐handed	90.6
	Left‐handed	7.5
	Ambidextrous	1.9
Maternal educative level	Bachelor's degree	66
	Technical	24.5
	Secondary	9.4
	Primary	0
Paternal educative level	Bachelor's degree	58
	Technical	20
	Secondary	18
	Primary	4
Siblings	Only child	37.7
	One	49.1
	Two	9.4
	Three	0
	Four	3.8
Position with respect to siblings	First	19.35
	Second	25.8
	Third	4.8

**Table 2:** Mean and standard deviation of neuropsychological outcomes, percentage of correct answers in Egocentric and Allocentric Spatial Memory Tasks, and cortisol values in age groups

Age	5	6	7
Mean (Standard deviation)			
RIST	108.62 (11.59)	109.19 (13.02)	110.55 (15.09)
Geometric puzzles	16.95 (2.25)	18.86 (2.33)	23.30 (4.11)
Route finding	3.05 (2.16)	4.05 (3.10)	6.05 (2.64)
Egocentric A	9.05 (1.20)	9.43 (0.92)	9.40 (0.94)
Egocentric B	6.30 (2.10)	6.71 (2.23)	7.10 (2.07)
Allocentric Total	15.05 (5.69)	18.05 (4.17)	18.95 (4.92)
Allocentric Block 1	5.70 (2.36)	5.62 (2.59)	6.35 (2.62)
Allocentric Block 2	4.05 (3.33)	6.14 (2.33)	5.90 (2.63)
Allocentric Block 3	5.30 (2.57)	6.29 (1.92)	6.60 (1.98)
Cortisol (µg/dl)	0.14 (0.05)	0.18 (0.06)	0.16 (0.05)
Mean percentage of correct answers (%)			
Egocentric A	90.48	94.29	94
Egocentric B	63	67.14	71
Allocentric Total	62.95	75.17	78.95
Allocentric Block 1	71.85	70.26	79.37
Allocentric Block 2	50.62	76.19	73.7
Allocentric Block 3	66.25	75.23	82.5

**Table 3:** Mean and standard deviation of neuropsychological outcomes, percentage of correct answers in Egocentric and Allocentric Spatial Memory Tasks, and cortisol values in gender groups

	Boys	Girls
Mean (Standard deviation)		
RIST	112.38 (12.94)	106.40 (12.74)
Geometric puzzles	20.32 (3.97)	19.03 (3.96)
Route finding	5.19 (3.02)	3.53 (2.56)
Egocentric A	9.44 (0.91)	9.13 (1.13)
Egocentric B	6.97 (2.28)	6.43 (1.96)
Allocentric Total	18.52 (4.71)	16.17 (5.38)
Allocentric Block 1	6.03 (2.51)	5.73 (2.54)
Allocentric Block 2	6.10 (2.53)	4.63 (3.10)
Allocentric Block 3	6.32 (2.19)	5.80 (2.23)
Cortisol (µg/dl)	0.17 (0.05)	0.16 (0.06)
Mean percentage of correct answers		
Egocentric A	94.38	91.33
Egocentric B	69.68	64.33
Allocentric Total	77.14	67.34
Allocentric Block 1	75.40	72.08
Allocentric Block 2	75.80	57.91
Allocentric Block 3	77.41	71.83

### 3.2 Spatial orientation and visuospatial ability performance

**Figure 3:**
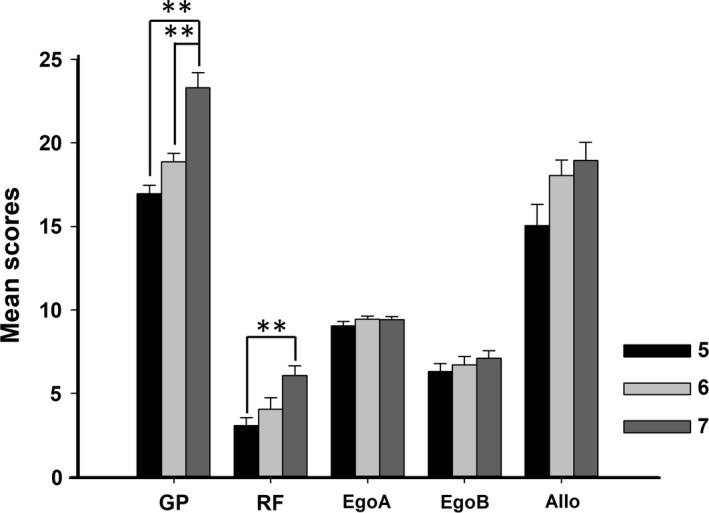
Visuospatial and spatial orientation outcomes in children of 5, 6, and 7 years. Significant differences were found in GP between 5 and 7 years and between 6 and 7 years. GP Geometric puzzles, RF Route finding, EgoA Egocentric Part A, EgoB Egocentric Part B, Allo Allocentric. **p < .01

First, we aimed to probe whether there are age‐ and gender‐related differences in visuospatial skills and spatial orientation. ANOVA (Age × Gender) disclosed a significant main effect of Age in Geometric Puzzles (*F*_2,55_ = 21.063, *p* < .001, *d* = 0.434) and Route Finding (*F*_2,55_ = 4.335, *p* = .018, *d* = 0.136). However, correcting by FDR differences is still significative in Geometric puzzles (*p* < .001), but not in Route Finding (*p* = .053). Egocentric Part A (*F*_2,55_ = 0.722, *p* = .490), Egocentric Part B (*F*_2,55_ = 0.255, *p* = .776), and Allocentric (*F*_2,55_ = 2.366, *p* = .103) tasks do not show significant differences according to age. No significant differences were found in any variable regarding Gender nor Age × Gender. The Tukey post hoc analysis revealed age differences in Geometric Puzzles between 5 and 7 (*p* < .001) and between 6 and 7 (*p* < .001) (Figure 3).

**Figure 4:**
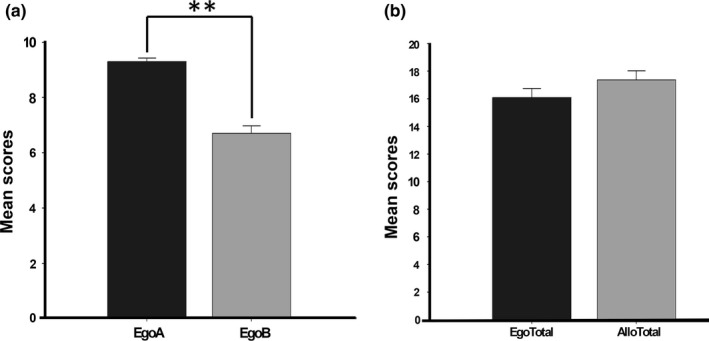
Comparison of Spatial Memory Tests. (a) Contrast between Egocentric part A and part B. Significant differences were found between Part A and B. (b) Comparison between Egocentric and Allocentric Spatial Memory Tests. No significant difference was found between frameworks. EgoA Egocentric Part A, Ego B Egocentric Part B, Ego Total Egocentric Total, Allo Total Allocentric Total. **p < .01

Secondly, comparing performance within the spatial orientation tasks, repeated‐measures ANOVA (Allocentric block 1 × 2 × 3) did not show significant differences between blocks in the whole sample (*F*_2,59_ = 1.459, *p* = .241), nor in the interaction with age (Allocentric blocks × Age) (*F*_4,110_ = 0.789, *p* = .538), nor in interaction with gender (Allocentric blocks × Gender) (*F*_2,54_ = 0.592, *p* = .557), nor in interaction with both age and gender (*F*_4,110_ = 0.642, *p* = .169). Paired *t* tests did not reveal significant differences between Egocentric and Allocentric test comparison in the whole sample (*t*_59_ = 1.588, *p* = .118), but we did find significant results contrasting Egocentric part A and Egocentric part B (*t*_59_ = 9.523, *p* < .001) (Figure 4).

Thirdly, we wanted to verify whether there is any association between spatial orientation, cognition, and visuospatial abilities. The Pearson correlation analysis showed a significant and direct relationship between several tasks (Table 4). The RIST test correlated with Route Finding (*r* = .280, *p* = .029) and Egocentric Part A (*r* = .264, *p* = .038). Geometric Puzzles correlated with Route Finding (*r* = .329, *p* = .010). Route Finding correlated with Egocentric Part A (*r* = .269, *p* = .036), Egocentric Part B (*r* = .404, *p* = .001), and Allocentric (*r* = .296, *p* = .022). Egocentric part A test correlated with Egocentric Part B (*r* = .262, *p* = .042).

**Table 4:** Correlation between neuropsychological tests in the whole sample

		RIST	Geometric puzzles	Route finding	Ego A	Ego B	Allo
RIST	Pearson Correlation	1	.107	**.280** *	**.264** *	.239	.168
	*p* value		.413	**.029**	**.038**	.064	.196
Geometric Puzzles	Pearson Correlation		1	**.329** **	.098	.118	.223
	*p* value			**.010**	.452	.370	.086
Route Finding	Pearson Correlation			1	**.269** *	**.404** **	**.296** *
	*p* value				**.036**	**.001**	**.022**
Ego A	Pearson Correlation				1	.251	.213
	*p* value					.051	.099
Ego B	Pearson Correlation					1	**.262** *
	*p* value						**.042**
Allo	Pearson Correlation						1
	*p* value						

Bold indicates statistically significant differences.

Abbreviations: Allo, Allocentric Spatial Memory Test; Ego A, Egocentric Spatial Memory Test Part A; Ego B, Egocentric Spatial Memory Test Part B.

* *p* ≤ .05

** *p* ≤ .01.

### 3.3 Spatial orientation and its relationship with cortisol, behavior, and memory in everyday contexts

The Pearson correlations were also used to assess the associations of the spatial orientation tasks with cortisol, with some other behavioral and psychological dimensions and with the use of spatial memory in daily life activities (Table 5). First, cortisol values are significatively related to Allocentric tests (*r* = .361, *p* = .024). Second, adaptive behaviors like leadership are significantly associated with Egocentric test Part A (*r* = .464, *p* = .003) and like adaptative abilities with Allocentric test (*r* = .277, *p* = .032). Some maladaptive behaviors like depression, atypicality, and hyperactivity are significantly and negatively related to Egocentric Part A (*r* = −.292, *p* = .022), Egocentric Part B (*r* = .319, *p* = .013), and Allocentric (*r* = −.273, *p* = .035), respectively. Finally, daily life memory assessed by ECM‐Q correlated with Egocentric Part A and Allocentric tests. Thus, Egocentric part A is significantly and positively associated with Item 1—Remember how to go home (*r* = .342, *p* = .014) and Item 7 (*r* = .439, *p* = .001) and significantly and negatively associated with Item 3—Forget how to go to a place (*r* = −.326, *p* = .022). The Allocentric test is significantly and directly related to Item 2—Good at orientation (*r* = .348, *p* = .012) and Item 4—Remember where things are (*r* = .340, *p* = .014), as well as, significantly and inversely related to Item 8—Get lost in previously visited places (*r* = −.423, *p* = .002).

**Table 5:** Correlation between Egocentric and Allocentric Spatial Memory Tasks with cortisol values, BASC measurements, and ECM‐Q questionnaire outcomes in the whole sample

		Ego A	Ego B	Allo
Cortisol (µg/dl)	Pearson Correlation	.010	.104	**.361** *
	*p* value	.950	.528	**.024**
BASC Aggressiveness	Pearson Correlation	−.026	.131	.199
	*p* value	.840	.318	.127
BASC Hyperactivity	Pearson Correlation	−.058	.049	**−.273** *
	*p* value	.658	.710	**.035**
BASC_Behavior problems	Pearson Correlation	.012	−.101	−.242
	*p* value	.944	.533	.133
BASC Attention problems	Pearson Correlation	−.207	−.222	−.071
	*p* value	.110	.089	.590
BASC Atypicality	Pearson Correlation	−.022	**−.318** *	.060
	*p* value	.864	**.013**	.651
BASC Depression	Pearson Correlation	**−.292** *	−.072	−.118
	*p* value	**.022**	.585	.371
BASC Anxiety	Pearson Correlation	−.095	.164	−.042
	*p* value	.465	.212	.752
BASC Shyness	Pearson Correlation	−.145	.002	−.012
	*p* value	.265	.990	.925
BASC Somatization	Pearson Correlation	.013	.054	.008
	*p* value	.921	.682	.952
BASC Adaptability	Pearson Correlation	.112	.040	−.089
	*p* value	.390	.760	.499
BASC Social skills	Pearson Correlation	.164	.228	.065
	*p* value	.208	.079	.621
BASC Leadership	Pearson Correlation	**.464** **	.227	−.247
	*p* value	**.003**	.158	.125
BASC Externalizing	Pearson Correlation	.070	.135	.213
	*p* value	.589	.305	.103
BASC Internalizing	Pearson Correlation	−.114	.116	.058
	*p* value	.383	.377	.658
BASC Adaptative abilities	Pearson Correlation	.239	.221	**.277** *
	*p* value	.064	.090	**.032**
ECM‐Q Item 1—Remember how to go home	Pearson Correlation	**.342** *	.130	.047
	*p* value	**.014**	.367	.744
ECM‐Q Item 2—Good at orientation	Pearson Correlation	.145	−.027	**.348** *
	*p* value	.304	.852	**.012**
ECM‐Q Item 3—Forget how to go to a place	Pearson Correlation	**−.326** *	−.149	−.190
	*p* value	**.022**	.312	.196
ECM‐Q Item 4—Remember where things are	Pearson Correlation	.168	.135	**.340** *
	*p* value	.229	.341	**.014**
ECM‐Q Item 5—Get lost in known places	Pearson Correlation	−.263	−.079	−.143
	*p* value	.063	.585	.321
ECM‐Q Item 6—Remember thing's place	Pearson Correlation	.191	.125	.133
	*p* value	.170	.376	.347
ECM‐Q Item 7—Recognize places	Pearson Correlation	**.439** **	.078	−.040
	*p* value	**.001**	.584	.776
ECM‐Q Item 8—Get lost in previously visited places	Pearson Correlation	−.204	−.057	**−.423** **
	*p* value	.150	.693	**.002**
ECM‐Q Item 9—Good at learning a new path	Pearson Correlation	−.011	.156	.254
	*p* value	.941	.279	.075

Bold indicates statistically significant differences.

Abbreviations: Allo, Allocentric Spatial Memory Test; BASC, Behavior Assessment System for Children; ECM‐Q, *Evaluación clínica de la Memoria;* Ego A, Egocentric Spatial Memory Test Part A; Ego B, Egocentric Spatial Memory Test Part B.

* *p* ≤ .05

** *p* ≤ .01.

## 4 DISCUSSION

The main purpose of this study was to assess egocentric and allocentric spatial orientation in typically developed children between the ages of 5 and 7, considering gender of the participants, and trying to elucidate how spatial orientation using these frameworks is associated with visuospatial skills, spatial daily memory, behavior pattern, and cortisol levels.

Firstly, we found that visuospatial abilities develop from 5 to 7 years of age. Concretely, mental rotation improves at 6 compared to 5, and at 7 compared to 6. Although mental rotation abilities start to develop very early in infancy, showing the very first signs at 6 months (Frick, Möhring, & Newcombe, 2014), it seems that mental rotation abilities really start to improve at the age of 3 (Kruger, 2018) and performance becomes steadier at the age of 5 (Frick, Ferrara, & Newcombe, 2013), but we have also verified that this development still continues at the age of 7, although until 10 children do not reach the same accuracy as adults in this ability (Wimmer, Robinson, & Doherty, 2017). Accurate visuospatial functioning and memory in regular development have been associated with number‐related skills and spatial processing (Cornu, Schiltz, Martin, & Hornung, 2018; Crollen & Noel, 2015) that finally could affect some learning abilities, mainly arithmetic accuracy and mathematical achievement (Foley, Vasilyeva, & Laski, 2017; Li & Geary, 2013, 2017).

Nevertheless, improvements are not found in egocentric and allocentric spatial orientation at these ages. The lack of progress in spatial orientation is contrary to previous results, where differences between the ages of 5 and 7 have been found in the allocentric framework (Bullens, Klugkist, & Postma, 2011; León et al., 2014; Mandolesi, Petrosini, Menghini, Addona, & Vicari, 2009), egocentric framework (Juan et al., 2014), and both frameworks (Nardini, Jones, Bedford, & Braddick, 2008). However, we found substantial methodological differences. Some of them jointly included older and younger age groups than ours (Juan et al., 2014; Mandolesi et al., 2009; Nardini et al., 2008) and some carried out the experiment in virtual environments (León et al., 2014), making it difficult to compare between ages and methods. Besides, it is noteworthy to point out that our limited sample size may also cause the absence of differences. Despite the lack of statistically significant results, previous studies also find small improvements specifically between the ages of 5, 6, and 7 in orientation skills (Piccardi et al., 2014; Rodriguez‐Andres et al., 2016), which are consistent with our descriptive results. Execution is almost the same between ages in Egocentric Part A and in Egocentric Part B, but there is slightly more of a marked difference between 5‐year‐olds compared to 6‐ and 7‐year‐olds in the Allocentric test. It is also worth mentioning that except for Egocentric part A, which reaches almost a ceiling effect, the rest of the tasks not. Therefore, it is possible that in later stages of development, the level of success in these tests could continue to increase. Besides, in order to minimize the influence of visuospatial span, only two items were employed in the present study. According to previous results, visuospatial span in an egocentric task, that is, the amount of visuospatial information the child has been able to memorize in his/her surrounding environment, is approximately two items at age 5, but 3 at age 7 (Piccardi et al., 2014). Therefore, two items can underestimate 7‐year‐old children's achievement.

Second, we do not find gender differences in any of the abilities measured. Starting with spatial orientation results, there was no gender effect found in several egocentric and allocentric spatial orientation tasks at the ages measured (Juan et al., 2014; Leplow et al., 2003; Piccardi et al., 2014; Rodriguez‐Andres et al., 2016). In methodologically equivalent tasks, it is found that in the young adult population, men outperform women in both frameworks (Fernandez‐Baizan Arias, & Mendez, 2019a, 2019b). Therefore, it is possible that gender differences frequently found in these tasks appear later in development, and so, it seems that the greatest differences appear from the age of 13 on (Nazareth, Huang, Voyer, & Newcombe, 2019). These results could be due, as the authors point out, to experiential and social norms associated with gender roles at these ages, where navigational behavior starts to be more independent (Nazareth et al., 2019), but also could be due to sexual hormone secretions (Driscoll, Hamilton, Yeo, Brooks, & Sutherland, 2005). Therefore, the beginning of puberty could be the developmental stage at which gender differences in spatial orientation start to be more marked.

There is no improvement in the Allocentric test performance in its different blocks, where children of these ages show a relatively homogeneous performance throughout the test. There is also no better performance of one framework over the other which seems to suggest that children at these ages perform the Egocentric and the Allocentric tasks with the same level of effectiveness. Reports have shown that while the egocentric framework emerges very early in development (Acredolo, 1978; Acredolo & Evans, 1980), the allocentric strategy reaches similar levels to adult performance between the ages of 7 and 10 (Leplow et al., 2003; Overman et al., 1996; Ruggiero et al., 2016). Thus, the absence of better performance of the egocentric framework over the allocentric may be due to several reasons. On the one hand, although the allocentric framework is not fully developed at these ages, children seem to prefer the use of allocentric landmarks rather than egocentric information (Yang, Merrill, & Wang, 2019). On the other hand, as mentioned above, the selection of a low number of items to memorize can make the task simple for older children. Besides, taking into account descriptive values, we can see how performance in the first block reaches high values, indicating that children are able to use an allocentric orientation response from the first trial, and therefore, a progressive learning effect is not observable. Possible future lines of research could be directed toward making the task more complex and to verify whether differences appear between the egocentric and allocentric framework when introducing greater difficulty.

However, we did find differences between Egocentric part A and B, with higher scores in the first part for all age groups. Part A serves as a 3D short‐term visuospatial memory measure, as well as a control measure for the second part. Part B is the one that measures egocentric orientation itself. Thus, the results found indicate that part A (span) is a good measure of control for the execution of part B (egocentric), knowing that in the first, a ceiling effect is practically expected. Thus, if a child fails to perform part A properly, we can expect that errors in part B are not due exclusively to a problem in egocentric orientation. Thus, there may be short‐term visuospatial memory difficulties affecting egocentric performance, or there may be both short‐term memory and egocentric orientation problems. In addition, these results are consistent with previous findings, where children perform better when their egocentric view remains stable, as opposed to when that view is rotated (Vander Heyden, Huizinga, Raijmakers, & Jolles, 2017).

Regarding the relationships between neuropsychological tasks, we observe that the most interrelated function is directionality and visual–spatial relationship establishment, measured by the Route Finding test. Thus, this ability is related to IQ (RIST), 3D visuospatial short‐term memory (Part A), egocentric (Part B), and allocentric orientation. Therefore, we can conclude that the execution of three‐dimensional spatial orientation tasks is partly influenced by the development of visuospatial skills, and vice versa. Mental rotation skills (Geometric Puzzles) are not related to the egocentric orientation (Part B). The first involves mental rotation of images, matching one figure with another one which has been rotated, while the second requires updating spatial information from a new position or a new view, after rotation of the participant's body position. This shows that the evaluation of traditional visuospatial skills (with pencil and paper tasks and in two dimensions) fails to measure all the capabilities involved in spatial cognition, and therefore, the inclusion of more functional and three‐dimensional measures would be adequate to have a full assessment of this ability in childhood. We also observed an association between 3D visuospatial short‐term memory skills (Egocentric Part A) and egocentric orientation (Egocentric Part B), although with a low magnitude. This result supports the idea, as we commented previously, that part A can be a good measure of control for part B. Finally, the absence of correlation between egocentric (Egocentric Part A and B) and allocentric orientation indicates that both tasks, indeed, measure fully dissociated abilities, as we know from their neuroanatomical substrates (Boccia, Nemmi, & Guariglia, 2014; Chen et al., 2014; Saj et al., 2014; Zaehle et al., 2007) and from neurological patients with hippocampal damage (Astur, Taylor, Mamelak, Philpott, & Sutherland, 2002), and therefore, the inclusion of both tests is necessary for a complete evaluation of spatial orientation abilities.

Finally, in regard to our findings, hormonal regulation of cortisol, behavior, as well as the spatial memory in daily life is related to spatial orientation results. First, high levels of salivary cortisol are related to better performance in allocentric framework. Thus, our results agree with those of Bohbot (Bohbot, Gupta, Banner, & Dahmani, 2011), where healthy adult subjects who presented higher levels of cortisol are those that use allocentric orientation more effectively. In this regard, although cortisol levels are associated with chronic stress and appear to affect hippocampal function over a prolonged period of time (McEwen & Sapolsky, 1995), it appears that occasional elevated levels of this hormone may favor better performance in allocentric orientation. To our knowledge, cortisol normative data in saliva for the ages and in the range of hours assessed in this study have not been published. In saliva samples, children between 9 and 12 years of age and with their samples recorded at 15:00 hr showed 0.16 µg/dl with a range of 0.07 and 0.33 (Catherine, Schonert‐Reichl, Hertzman, & Oberlander, 2012). In another study with samples collected at 20:00 hr, 6‐year‐old boys showed ranges between 0.076 and 0.612 µg/dl, 6‐year‐old girls between 0.076 and 0.336 µg/dl, 7‐year‐old boys between 0.043 and 0.893 µg/dl, and 7‐year‐old girls between 0.054 and 0.638 µg/dl (Törnhage & Alfvén, 2006). Analyzing our results (total range 0.054–0.317), our data would be within the values proposed by this last‐mentioned study. Despite this, it is still difficult to conclude that the cortisol levels of our sample are within the normative values according to circadian rhythms. We also must consider that these results are still preliminary and limited, and previous studies have seen that the reliable measure consists of a total diurnal salivary cortisol curve (Golden, Wand, Malhotra, Kamel, & Horton, 2011).

In terms of behavior, a child's behavior may have influence on spatial orientation functioning. More specifically, it seems that a better performance of 3D visuospatial short‐term memory (Egocentric Part A) would be found in children with fewer depression rates, as well as children with greater leadership skills. On the other hand, those children with more hyperactive behavior perform worse in allocentric orientation. Depressive symptoms have been related to several memory impairments, such us autobiographical (Kohler et al., 2015), prospective (McFarland & Vasterling, 2018), visuospatial (Gallagher, Gray, & Kessels, 2015; Klojcnik, Kavcic, & Bakracevic Vukman, 2017), and spatial (Han, Wang, Bian, Zhou, & Ruan, 2015; Lim et al., 2018). Hyperactive behavior has been related to memory deficits, concretely working memory, in ADHD disorders (Pievsky & McGrath, 2018). In addition, our allocentric test requires the child to move. Thus, although the examiner guides this movement, children who manifest a more active behavior are likely to move from the positions indicated, influencing the results obtained. Previous studies have linked the possible influence of behavior on spatial navigation tasks, finding that children who navigated faster scored higher on aggressiveness and that those children who scored higher on withdrawal and attention problems had more exploratory behavior (Rodriguez‐Andres, Mendez‐Lopez, Juan, & Perez‐Hernandez, 2018). These results highlight the importance of considering the influence of adaptive and maladaptive behavior on spatial orientation performance.

Memory functioning in everyday environments is related to spatial orientation performance. Egocentric A performance is related to items associated with short‐term memory and working memory, such as remembering a path, recognizing places previously visited, and forgetting the explanation of how to get to a place. Short‐term memory is involved in these processes, in the sense of spatial information maintenance. Working memory is associated with remembering how to get home and recognizing previously visited places. Although we consider that the Egocentric A is assessing spatial short‐term memory, as the task progresses it also begins to include a working memory component, because the child must inhibit the previously learned information to avoid interference. Regarding the allocentric test, we can observe that it mostly correlated with items directly related to navigation, learning, and following paths. Thus, allocentric tasks are related to a good spatial orientation of the child as perceived by the parents and with a low frequency of lost in known places, as well as with an appropriate memory of where the child leaves objects. It is also important to note that the results found in the allocentric test may include the ability to process egocentric information present during the resolution of the test. The Egocentric B task has not shown significant correlations with memory in everyday items. Thus, the evaluation of the egocentric orientation seems to work independently from the other tests and seems to indicate less functional relevance.

Several limitations are present in our study. The sample size analyzed is small. It is possible that these tasks are not sensitive to age or gender differences due to the level of complexity selected. Likewise, it is impossible to rule out the presence of egocentric information during the development of the allocentric task. Only the parents complete the questionnaire regarding behavior and memory in daily life. Other important observers of child's behavior, such as teachers, could give us a more complete view of these variables. Regarding cortisol, the use of a single measure has the lowest reliability compared to other protocols. Finally, some factors have not been considered in this research, such as the influence of academic performance and other cognitive functions, such as executive functions.

In spite of this, our study improves knowledge about typical development of egocentric and allocentric spatial orientation, based on differential and functional‐relevant tasks, in order to achieve a more complete view of visuospatial and spatial memory skills. This knowledge may allow the use of these tasks to detect possible alterations in these abilities in pathological populations with potential alterations of visuospatial abilities or certain behavioral problems, making possible early interventions. We have concluded that in normal development there are no marked improvements in egocentric orientation, allocentric orientation, or spatial relationship skills, but it does in mental rotation. We have also found that execution in spatial orientation may depend on other factors of physiological or behavioral origin, and, therefore, it may be important to consider their potential influence.

## CONFLICT OF INTEREST

All authors declare that there are no actual or potential conflicts of interest including any financial, personal, or other relationships with other people or organizations that could inappropriately influence this work.

## CONFLICT OF INTEREST

None declared.

## Data Availability

The data that support the findings of this study are available from the corresponding author upon reasonable request.
